# Rivaroxaban and other non-vitamin K antagonist oral anticoagulants in the emergency treatment of thromboembolism

**DOI:** 10.1186/1865-1380-6-25

**Published:** 2013-07-16

**Authors:** Patrick Goldstein, Ismaïl Elalamy, Kurt Huber, Nicolas Danchin, Eric Wiel

**Affiliations:** 1 Department of Emergency Medicine, CHRU de Lille, Lille, France; 2 Department of Haematology, Hôpital Tenon, Paris, France; 3 3rd Department of Internal Medicine, Cardiology and Emergency Medicine, Wilhelminenspital, Vienna, Austria; 4 Hôpital Européen Georges-Pompidou, Paris, France

**Keywords:** Acute thrombosis, Emergency setting, Oral anticoagulant, Pulmonary embolism

## Abstract

Pulmonary embolism (PE) is potentially fatal and often requires emergency management. Because PE associated with shock and/or hypotension carries a high risk of sudden death, emergency clinicians must rapidly make a diagnosis and initiate appropriate therapeutic strategies, usually involving anticoagulant treatment. Traditional anticoagulants, such as heparins and vitamin K antagonists, although effective and recommended by guidelines, are associated with limitations. Several targeted, orally administered anticoagulants that may overcome some of these constraints have been developed recently and undergone analysis in randomised, phase III clinical trials. Rivaroxaban, a direct factor Xa inhibitor, was non-inferior to standard therapy with enoxaparin plus a vitamin K antagonist for the prevention of recurrent, symptomatic venous thromboembolism (VTE) in patients with acute PE and led to a 50% reduction in major bleeding. Dabigatran, a direct thrombin inhibitor, was also non-inferior to standard therapy for the prevention of recurrent VTE or VTE-related death when given after a parenteral anticoagulant and had a similar incidence of major bleeding. The results of a phase III study of apixaban, another direct factor Xa inhibitor, for the acute treatment of VTE are expected in the near future. Rivaroxaban is now approved in Europe and the US for the treatment of acute PE and prevention of recurrent VTE. This article reviews the current guidance on the treatment of PE with special focus on the emergency setting, and considers data regarding rivaroxaban and the other non-vitamin K antagonist oral anticoagulants and their potential role, including patients who are and are not appropriate for treatment with these agents. Issues such as drug interactions, reversal of anticoagulant effect and coagulation monitoring are also discussed.

## Introduction

Pulmonary embolism (PE) is a potentially life-threatening emergency. In approximately 80% of cases, PE results from the embolisation of a deep venous thrombus [1,2]. Approximately 60% of patients with proven proximal deep vein thrombosis (DVT) also show evidence of PE, and the absence of DVT in a patient with PE could indicate that the fresh venous thrombus has already embolised [1,2]. Patients with PE have case fatality rates of 7-11% owing to the acute episode or a subsequent recurrence, which is higher than that associated with proximal DVT [3].

The standard antithrombotic treatment for DVT and PE is parenteral unfractionated heparin (UFH) or low molecular weight heparin (LMWH, e.g. enoxaparin), transitioning to an oral vitamin K antagonist (VKA) such as warfarin. Fondaparinux is a parenteral alternative to heparins. These agents are effective but have limitations, some of which may be overcome by the more recently developed non-VKA oral anticoagulants (OACs) [4]. Based on the phase III EINSTEIN trials, the direct factor Xa inhibitor rivaroxaban is now approved in Europe and the US for the treatment of DVT and PE as well as the prevention of recurrent DVT and PE in adults [5,6]. In addition, treatment studies involving the direct thrombin inhibitor dabigatran have also been completed, and studies of the direct factor Xa inhibitors apixaban and edoxaban are ongoing. This review describes the current guidelines for the treatment of patients with acute PE and considers the potential role of non-VKA OACs in this paradigm.

## Review

### Methods

Literature sources for this article were retrieved via PubMed based on a series of searches using the names of anticoagulant classes and specific generic names of the agents discussed herein, in various combinations with Boolean operators and the terms ‘deep vein thrombosis’, ‘pulmonary embolism’, ‘venous thromboembolism’ and ‘treatment’. Articles returned by these searches were reviewed for relevance to the subject of this review, and the reference lists of other pertinent review articles and guidelines documents were examined for additional sources of information, such as congress abstracts.

### Current guidelines on the emergency treatment of pulmonary embolism

The most recent international guidelines for the treatment of venous thromboembolism (VTE) were released by the American College of Chest Physicians (ACCP) in 2012 [7]. In Europe, the current European Society of Cardiology (ESC) recommendations covering PE were issued in 2008 [3] and do not include non-VKA OACs.

Patients with a high likelihood of PE should immediately receive effective anticoagulation, even pending the results of confirmatory diagnostic tests [3,7]. Severe or life-threatening PE associated with shock and/or hypotension should be treated with immediate intravenous UFH in combination with thrombolytic therapy or, in the case of contraindication or failure of thrombolysis, pulmonary embolectomy with concurrent haemodynamic and respiratory support (Table 1) [3,7]. If the thrombotic episode is acute but not considered immediately life threatening, i.e. suspected PE not associated with haemodynamic instability, anticoagulant therapy should be given. Initial parenteral anticoagulation (LMWH or fondaparinux rather than UFH) or oral rivaroxaban are recommended (Table 1) [7]. When a parenteral agent is used initially, a VKA should be started concurrently and the parenteral agent discontinued after a minimum of 5 days’ treatment or when the international normalised ratio (INR) stabilises within the target range of 2.0-3.0 for 2 consecutive days [3,7]. In contrast, rivaroxaban is given alone at a dose of 15 mg twice daily for 3 weeks followed by 20 mg once daily for the remainder of treatment [5,6].

**Table 1:** **Current guidelines for the initial treatment of acute PE **[3,7]

**Diagnosis**	**Recommendation**	**Grade of recommendation/level of evidence**
High-risk PE (with hypotension and/or cardiogenic shock)	Thrombolytic therapy (for patients who do not have a high risk of bleeding)	2C (ACCP)/1A (ESC)
	Accompanied by immediate i.v. UFH	1A (ESC)
Non-high-risk PE, including suspected PE	Initial parenteral anticoagulation or rivaroxaban*LMWH or fondaparinux preferred over:	1B (ACCP)/1A (ESC)
	i.v. UFH	2B/C (ACCP)/1A (ESC)
	s.c. UFH	2B/C (ACCP)/1A (ESC)
	For patients at high risk of bleeding and those with severe renal dysfunction, UFH with an aPTT target range of 1.5-2.5 times the normal range	1C (ESC)
	Initial treatment with UFH, LMWH or fondaparinux should continue for at least 5 days, with concurrent VKA started as soon as possible	1B (ACCP)/1A (ESC)
	Parenteral treatment to be discontinued after achieving target INR levels for at least 2 consecutive days	1B (ACCP)/1C (ESC)

*ACCP guidelines only; no grade of recommendation or level of evidence specified. *ACCP* American College of Chest Physicians, *aPTT* activated partial thromboplastin time, *ESC* European Society of Cardiology, *INR* international normalised ratio, *i.v.* intravenous, *LMWH* low molecular weight heparin, *PE* pulmonary embolism, *s.c.* subcutaneous, *UFH* unfractionated heparin, *VKA* vitamin K antagonist.

### Established anticoagulants for the treatment of pulmonary embolism

UFH, given either subcutaneously or intravenously, binds to antithrombin to catalyse the inactivation of serine proteases involved in the coagulation cascade [8]. However, binding to other plasma proteins also leads to unpredictable pharmacokinetics and pharmacodynamics, necessitating close biological monitoring and regular dose adjustments, and may induce side effects including heparin-induced thrombocytopenia (HIT) [8]. LMWHs, which are also administered parenterally as weight-based doses, have a longer half-life than UFH and are more specific for antithrombin, making them more predictable and associated with fewer non-haemorrhagic side effects [8]. As a result, LMWHs have largely replaced UFH, except in certain circumstances such as in patients with severe renal impairment [creatinine clearance (CrCl) < 30 ml/min], in which UFH is preferred because it does not accumulate in patients with compromised renal function (Table 1) [7]. In the randomised COLUMBUS study, which included more than 1,000 patients, LMWH (sodium reviparin) given as treatment for symptomatic VTE for 12 weeks in combination with VKA was as effective as UFH/VKA (incidence of VTE recurrence 5.3% vs. 4.9%; absolute difference of 0.4 percentage points indicating equivalence by the pre-defined criteria), with no difference in major bleeding (3.1% vs. 2.3%; *P* = 0.63) or mortality (7.1% vs. 7.6%; *P* = 0.89) [9].

The parenteral indirect factor Xa inhibitor fondaparinux is a synthetic pentasaccharide that is more specific for antithrombin than LMWH and has a longer half-life. It does not require routine monitoring and is not associated with a risk of HIT [8]. In a randomised phase III study in patients with symptomatic PE, fondaparinux was non-inferior to UFH for prevention of recurrent VTE when both were given for initial anticoagulation before transition to a VKA [3.8% incidence of VTE recurrence at 3 months with fondaparinux vs. 5.0% with UFH; absolute difference −1.2% (95% confidence interval −3.0% to 0.5%)]; the incidence of major bleeding was similar for both treatments (1.3% vs. 1.1%, respectively) [10]. A similarly designed study including patients with DVT reported equivalent outcomes between fondaparinux and enoxaparin for efficacy [3.9% incidence of recurrent VTE at 3 months vs. 4.1%, respectively; absolute difference −0.15% (95% confidence interval −1.8% to 1.5%)] and major bleeding (1.1% vs. 1.2%, respectively) [11].

Initial parenteral anticoagulation provides a swift onset of action, which is important in acute thrombosis treatment. On the other hand, a potential for overdose exists owing to the long half-lives of LMWH and fondaparinux, and although protamine sulphate is able to partially neutralise LMWH, no reversal agent exists for fondaparinux [4]. For longer term prevention of recurrent thrombosis, oral VKAs have traditionally been prescribed to patients when they leave hospital. VKAs do not induce HIT, but they are associated with various other problems. These include numerous food and drug interactions that lead to unpredictable pharmacokinetics and pharmacodynamics, which in turn necessitate regular coagulation monitoring and repeated dose adjustment [4].

### Rivaroxaban and other non-vitamin K antagonist oral anticoagulants for the treatment of pulmonary embolism

The direct factor Xa inhibitors rivaroxaban, apixaban and edoxaban, and the direct thrombin inhibitor dabigatran, have been developed to address the limitations of traditional anticoagulants. They are administered orally, have a fast onset of action, and their predictable pharmacokinetics and pharmacodynamics across a broad range of patients allow for fixed doses without routine coagulation monitoring [4]. Currently, rivaroxaban is the only such agent to be approved in Europe and the US for the treatment of DVT and PE and the prevention of recurrent DVT and PE, at an initial dose of 15 mg twice daily for 3 weeks followed by 20 mg once daily for an individualised duration of anticoagulation [5,6].

Rivaroxaban was evaluated for the treatment of PE in the phase III EINSTEIN PE study (Table 2), which was an open-label, non-inferiority trial comparing oral rivaroxaban (15 mg twice daily for 3 weeks, then 20 mg once daily) with standard subcutaneous enoxaparin (1 mg/kg) twice daily overlapping with a VKA for the treatment of symptomatic PE (with or without DVT) [12]. Treatment continued for 3, 6 or 12 months based on each patient’s benefit-risk profile, local standards and the investigator’s clinical judgement [12]. A parallel study in patients with DVT (EINSTEIN DVT), which had the same design but recruited patients with symptomatic acute proximal DVT (without PE), was also conducted (Table 2) [13]. The rationale for conducting separate DVT and PE studies, a unique approach among trials of non-VKA OACs, was the distinct nature of these conditions in terms of clinical management and expected patient outcomes. Considering that DVT is more common [14], a study including both conditions could be insufficiently powered to confirm efficacy for PE. The two-stage rivaroxaban dosing regimen was chosen after phase II dose-finding studies showed an improvement in asymptomatic thrombotic burden with twice-daily dosing in the acute phase of treatment but a better bleeding profile with once-daily dosing [15,16]. Given the potential for early recurrent thrombosis [17,18], this regimen maintains intensive anticoagulation in the acute phase and a balance between effective anticoagulation and bleeding risk thereafter [19].

**Table 2:** Principal outcomes of phase III studies of non-VKA OACs for acute VTE treatment

**Study name**	**Interventions**	**Patients**	**Treatment duration**	**Primary efficacy outcome**	**Bleeding outcomes**
EINSTEIN PE [12]	Oral rivaroxaban 15 mg bid for 3 weeks then 20 mg od vs. enoxaparin s.c. 1.0 mg/kg bid for ≥ 5 days plus VKA started ≤ 48 h after randomisation	≥ 18 years with confirmed acute symptomatic PE with or without DVT (*n* = 4,832)	3, 6 or 12 months	Symptomatic, recurrent VTE: 2.1% vs. 1.8% (*P* = 0.003 for non-inferiority)	Major or non-major clinically relevant bleeding: 10.3% vs. 11.4% (*P* = 0.23)
					Major bleeding: 1.1% vs. 2.2% (*P* = 0.003)
EINSTEIN DVT [13]	As for EINSTEIN PE	≥ 18 years with confirmed proximal DVT without symptomatic PE (*n* = 3,449)	3, 6 or 12 months	Symptomatic, recurrent VTE: 2.1% vs. 3.0% (*P* < 0.001 for non-inferiority)	Major and non-major clinically relevant bleeding: 8.1% vs. 8.1% (*P* = 0.77)
					Major bleeding: 0.8% vs. 1.2% (*P* = 0.21)
RE-COVER [20]	Parenteral anticoagulant indication then oral dabigatran etexilate 150 mg bid vs. oral warfarin (INR 2.0-3.0) od	≥ 18 years with acute, symptomatic VTE (*n* = 2,564)	6 months	Symptomatic VTE or VTE-related death: 2.4% vs. 2.1% (*P* < 0.001 for non-inferiority)	Major or non-major clinically relevant bleeding: 5.6% vs. 8.8% (*P* = 0.002)
					Major bleeding: 1.6% vs. 1.9% (HR = 0.82; 95% CI 0.45-1.48)
RE-COVER II [21]	As for RE-COVER	≥ 18 years with acute, symptomatic VTE (*n* = 2,568)	6 months	Symptomatic VTE or VTE-related death: 2.4% vs. 2.2% (*P* < 0.0001 for non-inferiority)	Any bleeding: 200 vs. 285 patients (HR = 0.67; 95% CI 0.56-0.81)
					Major bleeding: 15 vs. 22 patients (HR = 0.69; 95% CI 0.36-1.32)

*bid* Twice daily, *CI*, Confidence interval; *DVT*, Deep vein thrombosis; *HR*, Hazard ratio; *OAC*, Oral anticoagulant; *od*, Once daily; *PE*, Pulmonary embolism; *s.c.*, Subcutaneous; *VKA*, Vitamin K antagonist, *VTE*, Venous thromboembolism.

**Figure 1:**
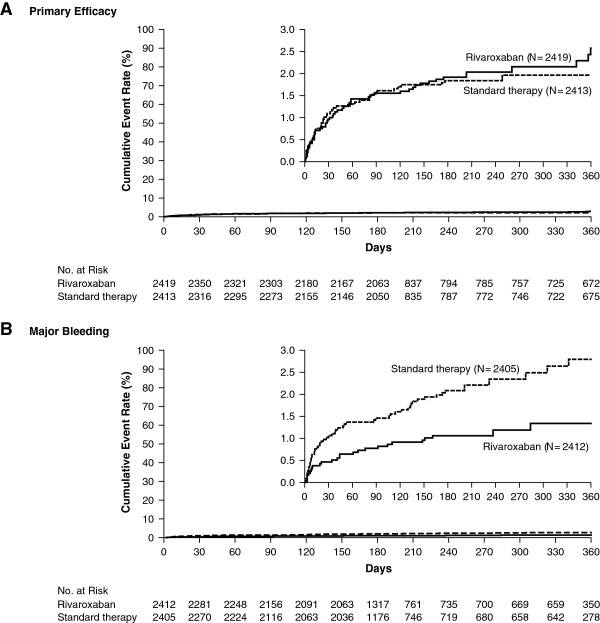
**Kaplan-Meier curves for the principal efficacy and major bleeding outcomes in EINSTEIN PE.** Incidences of **(A)** recurrent, symptomatic venous thromboembolism and **(B)** major bleeding with rivaroxaban versus enoxaparin/vitamin K antagonist [12]. From The New England Journal of Medicine: The EINSTEIN-PE Investigators, “Oral Rivaroxaban for the Treatment of Symptomatic Pulmonary Embolism”, volume 366, pages 1287–1297. Copyright © 2012, Massachusetts Medical Society. Reprinted with permission from Massachusetts Medical Society.

In EINSTEIN PE (*n* = 4,832), approximately one-quarter of patients with PE had concomitant DVT and approximately 65% had unprovoked thromboses [12]. Approximately 12% of patients in EINSTEIN PE were admitted to intensive care and approximately 24% had anatomically extensive PE [12]. In EINSTEIN PE, rivaroxaban was non-inferior to enoxaparin/VKA for the primary efficacy endpoint of recurrent, symptomatic VTE, with incidences of 2.1% versus 1.8%, respectively (*P* = 0.003 for non-inferiority; Figure 1A) [12]. The principal safety outcome, major plus non-major clinically relevant bleeding, occurred with a similar frequency in both study arms (10.3% vs. 11.4%, respectively; *P =* 0.23) [12]. There was no difference in the incidence of recurrent PE (32 vs. 27 cases) or death during the intended treatment period (2.4% vs. 2.1%). Significantly, in EINSTEIN PE, major bleeding, including intracranial and critical-site bleeding, was halved with rivaroxaban (1.1% vs. 2.2%; *P =* 0.003; Figure 1B) [12]. In EINSTEIN DVT (*n* = 3,449), rivaroxaban was also non-inferior to enoxaparin/VKA (2.1% vs. 3.0%; *P <* 0.001 for non-inferiority) with a similar incidence of clinically relevant bleeding between the treatment arms (8.1% vs. 8.1%; *P =* 0.77) [13].

**Figure 2:**
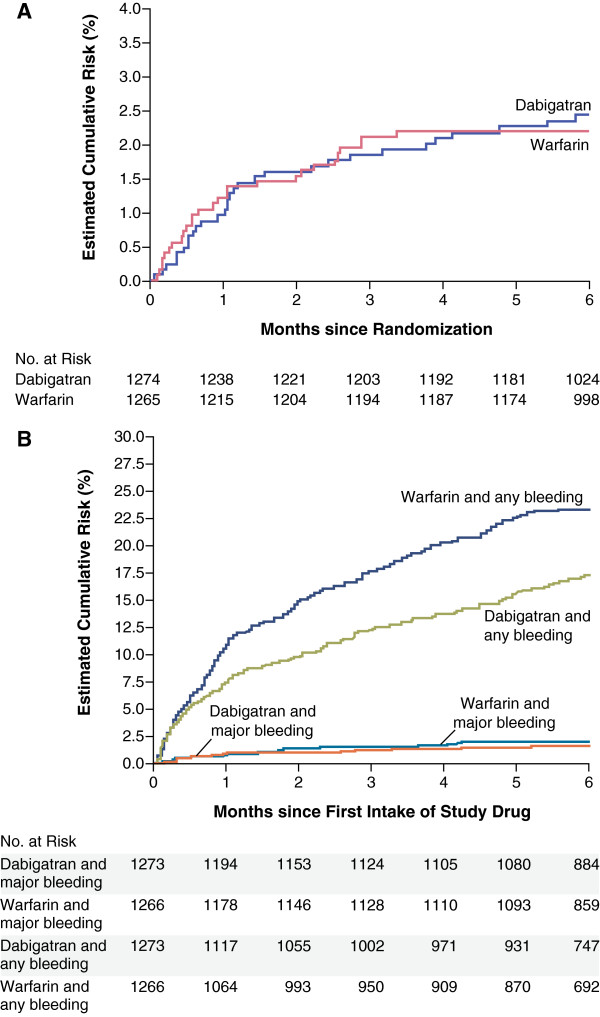
**Kaplan-Meier curves for the principal efficacy and bleeding outcomes in the RE-COVER study.** Incidences of **(A)** recurrent, symptomatic venous thromboembolism or related death and **(B)** major bleeding and any bleeding with dabigatran versus warfarin [20]. From The New England Journal of Medicine: Schulman et al., “Dabigatran versus Warfarin in the Treatment of Acute Venous Thromboembolism”, volume 361, pages 2342–2352. Copyright © 2009, Massachusetts Medical Society. Reprinted with permission from Massachusetts Medical Society.

Randomised, phase III acute treatment trials of apixaban, edoxaban and dabigatran have not assessed DVT and PE episodes separately. The majority of these are still ongoing but two studies – RE-COVER and RE-COVER II – comparing dabigatran etexilate 150 mg twice daily with warfarin for the 6-month treatment of acute, symptomatic VTE have been completed (Table 2) [20,21]. In contrast with the EINSTEIN trials, which used a single-drug approach from the start, patients in both study arms received parenteral anticoagulant induction. In both trials, which mostly enrolled patients with DVT, the two study arms were equally efficacious. In RE-COVER, the incidence of recurrent VTE or VTE-related death was 2.4% compared with 2.1% with warfarin (*P* < 0.001 for non-inferiority; Figure 2A), whereas in RE-COVER II, the same endpoint was associated with incidences of 2.4% and 2.2%, respectively (*P* < 0.0001 for non-inferiority). Rates of any bleeding and major bleeding were similar between the study arms in both RE-COVER (Figure 2B) and RE-COVER II [20,21]. A meta-analysis of published phase II and III studies found that, overall, the non-VKA OACs had equivalent efficacy in terms of preventing VTE recurrence compared with VKAs, but rivaroxaban was the only agent to significantly reduce the risk of major bleeding compared with standard care [22].

### Considerations for clinical practice in the emergency department

With a rapid onset of action, parenteral agents represent the routine choice for initial anticoagulation, but non-VKA OACs also show promise in this setting. Rivaroxaban is a specific factor Xa-targeting anticoagulant that provides a comparable speed of onset to enoxaparin [5,23], but without the risk of heparin-induced side effects [4]. Unlike the indirect factor Xa inhibitor fondaparinux, rivaroxaban is administered orally, binds directly to factor Xa without the need for antithrombin [24], and inhibits free, prothrombinase-associated and clot-associated factor Xa [25]. As the only non-VKA OAC currently approved for the treatment of DVT and PE, the following observations apply specifically to rivaroxaban, but some may also be relevant to non-VKA OACs as a class.

Overall, the EINSTEIN trials indicate that the risk of bleeding is similar between single-agent rivaroxaban and dual-drug LMWH/VKA. In EINSTEIN PE, there was no difference in the incidence of clinically relevant bleeding between the treatment arms in the acute phase of treatment (the first 21 days during which rivaroxaban was given at 15 mg twice daily), and, overall, major bleeding episodes were 50% less frequent with rivaroxaban [12]. Nevertheless, all anticoagulants confer a risk of bleeding, and it is important to pay attention to any clinical sign of bleeding. If appropriate, laboratory testing of haemoglobin/haematocrit and comparison with baseline values could indicate possible occult bleeding [5,26]. Anticoagulated patients who are at increased risk of bleeding, such as those with current or historical bleeding disorders, arterial hypertension, gastrointestinal ulcerations, vascular abnormalities or recent central nervous system trauma, should be monitored carefully for a drop in haemoglobin or blood pressure that may indicate bleeding. As with all anticoagulants, rivaroxaban should not be given to haemodynamically unstable patients, those who require pulmonary embolectomy or those receiving fibrinolytic agents [5,26].

Rivaroxaban is contraindicated in patients with active bleeding of clinical significance or a relevant likelihood of such bleeding caused by hepatic disease associated with coagulopathy, including cirrhotic patients classified as Child-Pugh B or C [5]. Approximately one-third of the rivaroxaban dose is excreted renally as unchanged drug and a further third is metabolised to inactive metabolites and then excreted renally [27,28]. No dose adjustment is generally required in patients with reduced renal function in the DVT/PE treatment setting, although caution should be applied in patients with CrCl 15–29 ml/min [5]. It should be noted that patients with CrCl <30 ml/min were excluded from the EINSTEIN studies [12,13]. Rivaroxaban is not recommended for patients with CrCl <15 ml/min, prosthetic heart valves or immediately life-threatening PE requiring thrombolysis or surgery (these patients were excluded from EINSTEIN PE). Only limited data are available on rivaroxaban use in cancer patients, who are generally at increased risk of both VTE and bleeding [7], but outcomes for rivaroxaban in the EINSTEIN trials for those patients with active cancer were generally consistent with results for the overall populations [12,13].

Concomitant administration of rivaroxaban with strong competitive inhibitors of cytochrome P450 3A4 and P-glycoprotein (P-gp) – namely azole-antimycotics or HIV protease inhibitors, including ritonavir – should be avoided [5,27]. Caution should be exercised when giving rivaroxaban to patients with moderate renal impairment (CrCl 30–49 ml/min) who are taking co-medications that could increase the risk of bleeding, such as antiplatelet agents, or could lead to a potential increase in rivaroxaban plasma concentrations (strong inhibitors of cytochrome P450 3A4 or P-gp, or moderate inhibitors of both). Conversely, strong P-gp inducers (e.g. rifampicin) could potentially cause an inadequate level of anticoagulation [5,27]. Co-administration of rivaroxaban with other anticoagulants is contraindicated unless switching to or from rivaroxaban, and use of rivaroxaban in combination with dronedarone should be avoided [5]. Some of these issues can be managed using clinical judgement or an alternative agent. However, a substantial proportion of patients are likely to be suitable for rivaroxaban because it has shown predictable properties regardless of demographic factors [29-33].

Because rivaroxaban has a relatively short half-life (5–13 h for a 10-mg dose) [5,34], reversal of anticoagulation should not normally be required with rivaroxaban. There is currently no specific antidote to rivaroxaban [35], although the use of activated charcoal could be considered in the case of recent overdose. Nevertheless, administration of up to 600 mg rivaroxaban has been reported without an increase in bleeding [5]. In the case of bleeding complications, rivaroxaban should be discontinued or delayed. Symptomatic treatment, such as mechanical compression, surgical haemostasis, fluid replacement and blood products, should be used in an individualised manner according to the nature and location of the bleeding. If these measures do not control bleeding, prothrombin complex concentrate, activated prothrombin complex concentrate or recombinant factor VIIa can be considered to reverse the anticoagulant effect, based on limited data [5,26]. Protamine sulphate and vitamin K infusion will not affect rivaroxaban activity, and there is no experience with antifibrinolytic agents or systemic haemostatic drugs. Rivaroxaban is not dialysable [5].

Rivaroxaban, like the other non-VKA OACs, has predictable pharmacokinetic and pharmacodynamic properties [29-33], and coagulation monitoring is not routinely necessary. A specific calibrated anti-factor Xa chromogenic assay is the preferred method for measuring rivaroxaban plasma concentrations (and those of other factor Xa inhibitors) if required in an emergency [26,36], but if unavailable, prothrombin time could be used. In the latter case, the reading must be made in seconds and compared against known rivaroxaban calibrators and controls; it must not be converted to an INR [5]. These tests are proposed to rule out the presence of the drug before acute surgery or to confirm neutralisation. If emergency surgery is required, rivaroxaban should ideally be stopped at least 24 h before the intervention, taking into account the patient’s risk of bleeding against the potential benefit of surgery [5]. Because of its relatively short half-life [5,34], rivaroxaban can be stopped closer to the time of surgery than VKAs and then be restarted when post-surgical haemostasis is established. Because of the rapid onset of effect with rivaroxaban, and in contrast to VKAs, there is no need for LMWH bridging.

The emergency physician may be faced with a patient with atrial fibrillation, or one who has recently undergone major orthopaedic surgery, who is taking anticoagulants but has symptoms suggestive of an acute PE. Poor control or non-adherence to VKA therapy could increase such a possibility. There is no official recommendation for this situation, but if the patient is already receiving rivaroxaban the logical strategy would be to increase the anticoagulant dose to an appropriate level for VTE treatment, provided the patient’s benefit-risk profile for recurrent VTE, atrial fibrillation-related stroke and bleeding is felt to be in favour of such an approach. The patient could be receiving dabigatran, but because dabigatran is not currently indicated for VTE treatment, a transition to an alternative agent may be necessary in this case. It has been suggested that rivaroxaban may be administered concurrently with acetylsalicylic acid (ASA), provided that the dose of ASA does not exceed 100 mg per day [37]. However, rivaroxaban 15 mg or 20 mg should not be combined with dual antiplatelet therapy (ASA plus clopidogrel, ticlopidine, prasugrel or ticagrelor) [37].

If a patient is receiving a VKA and control is suboptimal, switching to rivaroxaban for treatment is possible by discontinuing the VKA and starting rivaroxaban when the INR is below 2.5 [5]. There are circumstances in which oral anticoagulation is not possible or adequate in the emergency setting – for example, when the patient is unable to swallow a capsule or tablet. In this case, initial parenteral anticoagulation remains appropriate for induction therapy. It is then possible to switch to rivaroxaban by giving the first dose at the same time that the next dose of LMWH or fondaparinux is scheduled [5].

## Conclusion

Acute PE is a potentially life-threatening event that requires rapid, effective anticoagulant treatment. Non-VKA OACs, such as rivaroxaban, offer potential advantages over traditional antithrombotic agents for the treatment of VTE in many patients, simplifying their management without compromising antithrombotic efficacy or safety. Nevertheless, there are specific circumstances where these agents are not recommended, and clinical experience will serve to further define appropriate use.

## Abbreviations

ACCP: American College of Chest Physicians; ASA: Acetylsalicylic acid; CrCl: Creatinine clearance; DVT: Deep vein thrombosis; ESC: European Society of Cardiology; HIT: Heparin-induced thrombocytopenia; INR: International normalised ratio; LMWH: Low molecular weight heparin; OAC: Oral anticoagulant; PE: Pulmonary embolism; P-gp: P-glycoprotein; UFH: Unfractionated heparin; VKA: Vitamin K antagonist; VTE: Venous thromboembolism.

## Competing interests

**Financial competing interests:** This manuscript was developed with support from Bayer HealthCare Pharmaceuticals and Janssen Scientific Affairs, LLC. The article processing charge was paid by these parties. PG has received lecture and advisory board membership honoraria from Bayer HealthCare. IE and KH have received lecture fees from Bayer HealthCare. ND has received lecture or consulting fees from Bayer HealthCare. EW has no competing interests to declare. **Relevant stock or share ownership:** None. **Related patents:** None. **Other financial competing interests:** PG has received lecture and advisory board membership honoraria from Boehringer-Ingelheim, Daiichi Sankyo, Sanofi-aventis and The Medicines Company. IE has received lecture fees from Boehringer-Ingelheim, Bristol-Myers Squibb/Pfizer and Daiichi Sankyo. KH has received lecture fees from Boehringer-Ingelheim, Bristol-Myers Squibb/Pfizer, Daiichi Sankyo, Sanofi-aventis and The Medicines Company. ND has received research grants from AstraZeneca, Daiichi Sankyo, Eli Lilly, GlaxoSmithKline, Merck, Novartis, Pfizer, Sanofi-aventis, Servier and The Medicines Company and lecture or consulting fees from AstraZeneca, Bristol-Myers Squibb, Boehringer-Ingelheim, Daiichi Sankyo, Eli Lilly, GlaxoSmithKline, MSD-Schering, Novartis, Novo Nordisk, Pfizer, Roche, Sanofi-aventis, Servier and The Medicines Company. **Non-financial competing interests:** None.

## Authors’ contributions

All authors made substantive intellectual contributions to this manuscript and critically reviewed and revised the manuscript content. All authors have read and approved the final manuscript.
